# A Practical Treatise on Midwifery; Containing the Result of 16,654 Births, Occurring in the Dublin Lying-in Hospital, during a Period of Seven Years, Commencing November, 1826

**Published:** 1836-07

**Authors:** 


### ART. III.

*A Practical Treatise on Midwifery; containing the Result of* 16,654 *Births, occurring in the Dublin Lying-in Hospital, during a Period of Seven Years, commencing November*, 1826. By ROBERT COLLINS, M.D., late Master of the Institution.—*London*, 1836. 8vo. pp. 526.

A WORK like the present, the result of seven years' experience at the head of such an establishment as the Lying-in Hospital of Dublin, naturally excites much attention: it is looked upon as forming a sort of epoch in the annals of midwifery, and as enabling the author to lay before the professional world a complete system of practical obstetric science. Such a work is expected to be the great test of the recorded knowledge collected during the last half century, where, analysed by its author's immense experience, we are enabled to separate the really valuable matter from the dross, and thus condense the quantity of materials, which otherwise must unavoidably encumber and obscure our progress. It is to a careful and accurate record of the vast opportunities for observation enjoyed by those favoured individuals of our profession who are attached to similar institutions that we must look for the deciding of those facts, upon the establishment or refutation of which the improvement, and consequent success, of future practice must mainly depend.

It was with feelings of this kind that we opened Dr. Collins's review of his seven years' mastership at the Dublin Lying-in Hospital; and we may truly add, that they were accompanied by those of high respect and admiration, not only for his talented successor, but also for several other Dublin obstetricians, whose researches during the last few years have contributed so many valuable facts to our present stock of knowledge, and have gained for the Irish school of midwifery the high name and rank which it so deservedly bears at the present moment.

In a statistical point of view, Dr. Collins's work presents a mass of information unequalled by any preceding publication, and bears ample testimony to the care, industry, and patience of its author. In this respect alone it will always prove not only a valuable but also a *necessary* book of reference upon these subjects. We could wish that the whole statistical records of this magnificent institution were collected and embodied into one work; for we are well aware that materials exist to a great extent from an early period, the arrangement and publication of which would add immensely to their value. It is true that this object, to a certain degree, will be answered by the statistical commission established by the present government, the object of which, among other things, is to collect the records of all the great institutions in the united kingdom. These documents, however, can only be in the hands of a few; whereas, if published separately in the form of a book, they would be attainable by all.

In reviewing Dr. Collins's work, we propose to separate, as far as possible, the statistical from the practical part, condensing his extensive and elaborate records into a short and general view, in order to present some of the most important facts to our readers, without overstepping the limits to which we must necessarily confine ourselves.

16,434 women were confined at the Dublin Lying-in Hospital, during the seven years of Dr. Collins's mastership. These gave birth to 16,654 children; of which, 1,121 were still-born; and of these, 293 were premature.

240 cases of *twins* occurred; and, of the 480 children thus born, 422 were born alive; of these, 245 were males.

*Triplets* occurred four times: in two cases it was the patient's second, and the two others her third pregnancy.

The mean of the patients' ages was 29¾. All the children were born alive: one case was premature; eight are stated to have died.

The *face* presented in thirty-three cases; four children were still-born.

The *breech* presented in 242: of these, seventy-three children were still-born, forty-two putrid, and forty of the 242 premature.

The *feet* presented 137 times, (not including twins;) sixty-two were still-born, forty-one putrid, and thirty-six premature.

In forty cases the *shoulder or arm* presented; thirty-three children were turned; of which, twenty were born alive, six were putrid. In three of the thirty-three, the head required to be lessened.

Eleven cases of *placenta prævia* occurred; in eight of which, the child presented naturally, four were turned, one was expelled by the natural efforts, one was delivered by the forceps; in two the head was lessened; two presented with the feet, and one with the breech; six were born alive, two were putrid. Two of the women, in whom the children were turned, died.

*Hemorrhage after the expulsion of the placenta* took place in forty-three instances: viz. twenty cases of it during the first fifteen minutes, two in twenty minutes, one in thirty minutes, two in forty-five minutes, five during the first hour, two in one hour and a half, three in two hours, two in three hours, one in four hours, one in six hours, and one in twelve hours. In one case it took place on the fourth day, one on the fifth, and one on the tenth day. Four of these patients died: one from rupture of the uterus, one from sloughing of the vagina, and two from hemorrhage.

*Retention of the placenta* occurred in sixty-six cases: in thirty-seven it was from want of uterine action, in nineteen from spasmodic or irregular action, in ten the placenta was adherent; four were twin cases; in twenty-four there was slight hemorrhage; and in four the delivery had been "forced." Six of the sixty-six women died: viz. four of puerperal fever, which was then prevalent; one of inflammation of the uterus; and one, a feeble woman, sunk on the eighth day after delivery. In each of these six, the placenta was retained by irregular action of the uterus.

*Convulsions* appeared in thirty patients, of whom twenty-nine were primiparæ; the other patient was in her second pregnancy, and had suffered from a similar attack before. Fourteen of the thirty-two children (two cases of twins,) were born alive. In eighteen women the convulsions subsided after delivery; in ten they occurred both before and after; and in two the attack did not appear till after delivery. Fifteen were delivered by the natural efforts, six by the forceps, eight by perforation; in one case the feet presented. Five women died.

*Rupture of the uterus or vagina* took place in thirty-four instances: in thirteen posteriorly, in twelve anteriorly, in two laterally; in one the os uteri was torn; and in six the precise situation of the injury was not mentioned. In nine cases of the thirty-four, the peritoneum was not torn; in one, there were numerous lacerations of the peritoneum, without the substance of the uterus being torn. Two women recovered. The following scale shows the proportion of cases occurring in first, second, &c. labours.

| No. of Pregnancy, | 1 | 2 | 3 | 4 | 5 | 6 | 8 | 9 | 10 | 11 |
|---|---|---|---|---|---|---|---|---|---|---|
| No. of Women, ... | 7 | 6 | 6 | 2 | 2 | 5 | 1 | 1 | 2 | 2 |

The funis prolapsed in ninety-seven cases, in twenty-four of which the child was born alive. Twelve of the ninety-seven occurred in twin cases; seven of the twelve with the second child; nine where the feet presented, two where the breech, four where the shoulder or arm, and seven where the hand presented with the head. Seven children were putrid, and three premature.

One hundred and sixty-four women died, (one in one hundred.) In giving a scale of the deaths occurring in first, &c. pregnancies, the great proportion of deaths met with in first cases will at once strike the reader. "We should carefully bear in mind," says Dr. Collins, "that, of the 16,414 women, 4,969 gave birth to *first* children, which is nearly a *third* of the entire; therefore, any relative proportions should be made with reference to this fact."

| No. of Pregnancy, | 1 | 2 | 3 | 4 | 5 | 6 | 7 | 8 | 9 | 10 | 11 | 13 |
|---|---|---|---|---|---|---|---|---|---|---|---|---|
| No. of Women, ... | 86 | 20 | 11 | 11 | 9 | 7 | 2 | 6 | 2 | 3 | 2 | 1 |

The following table shows the causes of death in the above cases:

| | |
|---|---|
| Diarrhœa . . . . | 1 |
| Typhus fever . . . | 5 |
| Rupture of the uterus or vagina . | 32 |
| Uterine hemorrhage . . | 11 |
| Puerperal fever . . . | 59 |
| Inflammation of the brain . | 3 |
| Ulceration of the intestines . | 3 |
| Hectic fever . . . | 1 |
| Grief, apparently . . . | 2 |
| Stricture of intestine . . | 1 |
| Effects of tedious and difficult labour | 11 |
| Convulsions . . . | 2 |
| Sloughing of vagina . . | 6 |
| Pericarditis . . . | 1 |
| Peritoneal inflammation (placenta retained) . . . . | 4 |
| Abscesses in spinal canal . | 1 |
| Lumbar abscess . . . | 1 |
| Phthisis . . . | 2 |
| Diffuse cellular inflammation . | 1 |
| Abscess in abdomen . . | 2 |
| Acute bronchitis . . . | 1 |
| Anomalous disease . . | 12 |

Eighty-eight women were attacked with *puerperal fever.* In thirty-two cases it appeared on the first day; in twenty-nine, on the second; in eight, on the third; in two, on the fourth; and, in one case, on the eighth day. The mortality is stated above.

Of the 16,654 children which were born, 1,121 were still-born, 527 were putrid; 293 of the 1,121 were premature; 460 of the 1,121 were first children. For further particulars of children dying in the hospital, we must give the author's statement in his own words:

"The total number of children born was 16,654: of these, 284 died previous to the mother leaving the hospital. This is nearly in the proportion of one in 58½, which must be considered a moderate mortality under any circumstances: however, when it is considered that this includes not only *all the deaths that occurred in children born prematurely, and in twins, but also every instance where the heart even acted, or where respiration ceased in a few seconds after birth,* the proportion of deaths becomes *trifling* indeed."

Thirty-two of the children who died were twins, and, of these, seventeen were premature.

So much for the statistical portion of Dr. Collins's work. To enter more into the details of such a subject would be impossible; we must therefore refer the reader to the work itself. It is a lesson to all those connected with similar institutions, and shows the immense value of recording the particulars of every case under a proper arrangement.

Let us now examine the practical portion. Dr. Collins commences with some simple but very excellent "practical observations on Natural Labours:" he insists upon the importance of keeping the bowels open before labour, and on the utility of occasional enemata, &c. He cautions against examining too frequently in early stages of labour. With this we, of course, fully concur; but cannot agree with him where he says that no useful information can be thus obtained. Is it of no importance to ascertain the effect which the pains have on the os uteri, and thus decide whether the labour has really commenced?—whether she be a primipara, and, if so, whether the pelvis be well or ill shaped?—whether the head presents, and whether the funis or the hand is to be felt by the side of it?—whether the membranes remain unruptured, &c.? Is it of no interest, as well as consequence, to ascertain the precise position of the head, and carefully to watch its progress through the pelvis and external passages? To our surprise and sincere regret, we find the whole subject of natural labour passed over without one allusion to the subject of head-presentations, and without a single observation on the manner in which the head during labour enters and passes through the pelvis and external passages. We are at a loss to assign a reason for such an omission, especially in an institution where the recording of every case is attended to with so much accuracy and zeal, and where the memory of such a name as that of Fielding Ould ought to be cherished with the greatest respect. We look upon the carefully ascertaining the exact position of the head in *every* case where it presents, to be of the utmost importance: it not only leads to simple and correct views respecting the mechanism of parturition, but, from the degree of tact which it gives, we are enabled to detect any deviation from the natural course of labour, whenever it may occur. It is only in such institutions as this that we can have opportunities sufficient to come to great conclusions on these subjects; and we apprehend that there are few more deserving of attention than the mechanism of natural labour.

In speaking of the management of the perineum, Dr. Collins has not mentioned the danger to which it is exposed of rupturing when the shoulders pass, and which is decidedly one of the most frequent causes of severe perineal laceration.

He very properly (p. 6,) directs that the cord should be passed over the shoulders whenever it is twisted round the neck at all tightly; but, supposing it to be too tight even for this, and we have known it to be the case, what are we then to do?

In speaking of tedious and difficult labours, we are glad to find that the author deviates from the old and faulty custom of making the word forceps a plural substantive. He very correctly says the forceps *was* applied. He uses, with equal propriety, throughout his work, the word *pubes* instead of pubis. We could wish that he had not interspersed certain expressions which look strange to the eyes of his English brethren, such as *nurse-tender, force-delivered, stuping, hippo,* &c.: the two latter, to a person who has never been in Ireland, are perfectly unintelligible. The term "force-delivered," we presume, is taken from the *accouchement forcé* of the French authors, where, in consequence of profuse hemorrhage, convulsions, &c., the os uteri was forcibly dilated, and the child thus turned and extracted; a mode of practice entirely abandoned in modern times. Dr. Collins has not explained whether this expression refers also to *all* instrumental deliveries, or merely to cases of perforation.

In speaking of the forceps under the head of "Tedious and Difficult Labours," the author considers it (p. 11) not only an abuse of this instrument, but most hazardous to the patient, to apply it under any other circumstances than where "the os uteri is fully dilated, the soft parts relaxed, the head resting on the perineum or nearly so, and the pelvis of sufficient size to permit the attendant to reach the ear with the fingers." The first two conditions are necessary, the third is highly desirable, but we cannot agree with him as to the last condition. We are aware that in this respect we differ essentially from the general rule of English obstetricians, and consider the diagnosis of the position of the child's head by means of the ear in difficult labours requiring the forceps as liable to much objection. Whenever the head completely occupies the antero-posterior diameter of the pelvis, and presses firmly against the symphisis pubis, we hold it to be nearly if not quite impossible, at any rate highly improper, to attempt feeling for the ear; and yet many of these are cases where, although the soft parts are fully relaxed, the head is not *nearly* resting upon the perineum, although the great degree of cranial swelling might in some instances lead us to suppose it, and yet where the result has proved that the forceps *was* indicated, by the child being born alive, and the mother recovering without any injury.

Is the forceps merely an instrument of traction? Do we in nowise facilitate the delivery of the head by compressing it, and thus aiding nature in moulding it to the shape of the passages? We apprehend that one of its most important actions is that of applying pressure. We have, it is true, repeatedly seen the finger introduced, under these circumstances, in search of the ear; but it has been rarely, if ever, done without great complaint and suffering on the part of the patient.

It is here that the study of the sutures and fontanelles is so valuable, that a correct knowledge of the precise manner in which the head advances through the pelvis is, at least in our opinion, so necessary; and we must again express our surprise and regret that this interesting subject, the very basis of practical midwifery, should have been so entirely passed over in silence. "We would earnestly recommend," says Dr. Dewees, in his admirable System of Midwifery, § 64, "the study of the fontanelles and sutures to the beginner of the practice of midwifery, by early accustoming himself to touch and distinguish them: it will lead him with certainty to the situation of the head as regards the pelvis, &c." And, in the following section, he says, "Many rely upon the position of the ear for the knowledge of the situation of the head, but we very loudly object to this test, &c." We unquestionably agree with

Dr. Collins that it would be quite improper to apply the forceps in cases where the pelvis measured "little more than three inches from pubes to sacrum, and in others less than this:" we are not referring to such cases, but to those where the degree of pelvic contraction is very slight, or where the difficulty depends on the full size and development of the fœtal head: in such cases there is sufficient pressure to prevent the introduction, or rather *boring up* of the finger, (for we can call it nothing else,) behind the symphisis pubis, to search for the ear; and yet there is room enough in the oblique diameters to introduce the forceps, and, with patient and gentle traction and compression, at length to deliver the head without danger to the mother or child. The practice, against which he so justly exclaims, of dragging a child through a narrow pelvis, is still in vogue at Paris, where a forceps of great length and power is applied, and a degree of unfeeling violence used, which cannot be too strongly reprobated. The late Professor Osiander, of Göttingen, was, we believe, the only obstetrician of any name out of France who advocated a similar practice.

In speaking of Perforation, Dr. Collins passes a very high and just eulogium upon the value of the stethoscope in ascertáining whether the child be alive or dead, and, with the most honorable candour, confesses that formerly he must have occasionally committed error in delaying the delivery too long, from not being able to decide this important question. In the obstetric part of our last volume, we fully entered into the merits of this valuable instrument, and cordially join our author, in his note to page 21, in recommending the excellent work on this subject by the present master of the Dublin Hospital.

There is one observation (p. 17,) which struck us as requiring slight comment. In speaking of the difficulty of deciding as to the necessity of perforating, Dr. Collins says that, except where the misproportion between the child's head and pelvis is very great, "no individual can foretel whether the uterine action may be sufficient or not to expel the child; therefore, the most certain proof we have of such disproportion existing is the head remaining stationary for a number of hours after the dilatation of the mouth of the womb, uterine action during this time continuing strong." In the majority of cases of difficult labour arising from contracted pelvis, the os uteri does not dilate readily, but its edge remains thin and rigid for many hours; the pains do not appear to act in the proper direction, and this we presume, in some degree, results from the pressure to which the lower portions of the body of the uterus are subjected by being squeezed between the head and the pelvis. In many of these cases we should question the safety of waiting till the full dilatation of the os uteri. Where the patient is becoming exhausted,—where bleeding, laxatives, injections, opiates, &c. have been tried in the proper degree,—where the os uteri has dilated to about one and a half, or one and three quarter inches in diameter, we should proceed to lessen the head, feeling confident that, as soon as the injurious pressure was removed, the dilatation of the os uteri would proceed. These are not cases for the forceps; the state of the os uteri forbids it. They occur most frequently in primiparæ; and our not finding the head, at the commencement of labour, so low as is usually the case in first labours, ought instantly to suggest an apprehension of misproportion between the head and pelvis. The child rarely continues alive until the full dilatation of the os uteri, and, by thus delaying, we are running a great risk of those dangerous sloughings and severe injuries of the soft parts, against which the author so properly warns us.

Dr. Collins has not favored us with a description of the forceps used by himself and colleagues in the Dublin Lying-in Hospital; which would have been desirable.

The next chapter (and it is to be regretted that the chapters are not numbered,) consists of observations on "Presentations of the Face, and with the Face to the Pubes." It is little more than a statistical account: no notice is taken as to *how* the face presented, or of the manner in which it passes through the pelvis and external passages; which appears to us an important omission.

Twelve cases are said to have occurred where the face was turned to the pubes. Of these, six were still-born; four being delivered by the crotchet, and one being acephalous. It is to be lamented that the other cases of this presentation were not more accurately observed.

In the chapter on Breech-Presentations, the author tells us that "the breech of the child may be distinguished from any other part by its softness, the depression between the nates, the organs of generation, the anus, and the discharge of the meconium. Although this latter is sometimes observed when the head presents, in consequence of severe pressure, yet, under these circumstances, it comes away in a more fluid state, and has not its natural appearance, being mixed with the discharges from the uterus and vagina." But the breech is not to be distinguished by these signs, whether taken separately or conjointly: it is to be distinguished by the coccyx, with the sacrum above it, the anus below, and neither by the softness of its feel or the parts of generation. By such rules, how should we be able, when the presenting part is swollen by the pressure of the surrounding parts, to distinguish between the nates and the face. It would be nearly, if not quite, impossible. We have, however, already discussed this subject in our former volume, (p. 114.)

In the following page (41,) Dr. Collins very correctly observes, that in breech presentations, where there has been no interference in the previous part of labour, it is rarely necessary to turn the child with its occiput towards the pubes," the breech naturally making the turn above alluded to in its passage." This is a valuable observation, and we could wish that the fact were more generally known, as it would prevent much of that officious interference with nature which frequently renders breech cases so dangerous to the child's life.

It is not desirable to introduce the forefinger of the left hand into the mouth, as recommended by the author: it is much better to place two fingers on the upper jaw, where pressure may be safely applied.

Dr. Collins reckons breech cases among preternatural presentations, (p. 44;) but why should this be so? the child presents with the long axis of its body parallel to the axis of the superior aperture of the pelvis. What is there preternatural in a breech case? Among the breech cases which have been recorded, one child is stated to have weighed above thirteen pounds. These extraordinarily large children are by no means common, and are mostly still-born; in the present instance, however, the child was alive. Within the last few weeks, a case has been mentioned to us of a child which was born alive, at the City-Road Lying-in Hospital of London, weighing fourteen pounds; but the late Sir Richard Crofts is stated to have delivered a living child weighing fifteen pounds, which is quite enormous.

In cases of arm or shoulder presentation, (p. 66,) where the os uteri is not fully dilated, and is so rigid as to make it impossible to introduce the hand for the purpose of turning, we fear that the practice recommended is not so decided and effective as it ought to be. Dr. Collins says, "in such a situation, where the individual is strong and plethoric, twelve or fourteen ounces of blood should be taken from the arm, and a tablespoonful of the following mixture (an antimonial solution of one grain to one and a half ounce of water with acetum opii,) given every half hour." In such cases we never think of the quantity to be drawn, but of the effect to be produced: in a few weakly London-born females, the loss of fourteen ounces of blood has produced sufficient faintness to effect our object; but, in the majority of cases, especially among the poorer classes of Irish, any thing less than double that quantity has seldom sufficed. Dr. Dewees's observations on this subject are so admirably to the point, that we cannot do better than express our own views in his simple and impressive language. "Bloodletting," says this accomplished obstetrician, (§ 629,) "is the only remedy with which we are acquainted that has any decided control over the contracted uterus. It is one almost certain of rendering turning practicable under such circumstances, if carried to the extent it ought to be. A small bleeding in such cases is of no possible advantage; for, unless the practitioner means to carry the bleeding to its proper limit, which is the disposition to or actual state of syncope, he had better not employ it."

The directions for turning are scanty, although as far as they go, good: we must not, however, forget the quantity of matter which

Dr. Collins has had to condense into one volume, and make allowances accordingly. The diagnosis between the knee and elbow ought to have been mentioned, for it is very important in these cases.

The author (p. 71,) quotes the authority of Dr. Clarke, of Dublin, in showing the futility of attempting to turn putrid children which present wrong. His own directions for conducting embryulcia under these circumstances are very judicious, and too valuable to be passed by without quoting.

"Dr. Clarke, in his Report of the Dublin Lying-in Hospital, states that 'he had heard of several patients who lost their lives by practitioners of good repute insisting on turning the fœtus, though evidently putrid.' 'Would not,' he adds, 'a better chance be afforded to patients so situated, by perforating the thorax or abdomen, so as to lessen their bulk, and, by the aid of the crotchet or blunt hook, bringing down the breech?' This, from ample experience, is the practice we would unhesitatingly recommend; and that in all cases where its death could be satisfactorily ascertained. We have performed this operation repeatedly without the slightest injury to the patient, except in one instance, where the pelvis measured but two and a half inches from pubes to sacrum; nor do we think, where common caution is used, that there is, comparatively speaking, any risk to the patient. Delivery in this way is very troublesome, in most instances requiring an hour and a half or two hours for its completion. A free opening must be made with the ordinary perforator into the thorax, so as to permit us completely to empty it of its contents. We next open through the diaphragm, and remove the abdominal viscera, in order, as much as in our power, to diminish the bulk of the body. For this purpose the crotchet and fingers are to be used. We then fasten the crotchet on the pelvis of the child, and, giving gentle assistance with each pain, where the woman is well-formed, the breech, by a little perseverance, will be got down, and the delivery accomplished. Where we find much resistance, and there is no very urgent symptom rendering speedy delivery necessary, by withholding further assistance for some hours, the body becomes softened and collapsed, and is then more easily removed. In some instances the child is expelled doubled by the action of the womb."

We are surprised to find that, in an institution like this, where a head physician, assistant physicians, and also pupils, are constantly residing, it should not be a rule for every patient to be examined upon her admission by one of them, and the result of the examination properly entered in a book for that purpose, whether it be a common or uncommon case: were it only to ascertain the precise nature of the presentation, the value of such a record would be sufficient reason. In three of the cases of shoulder or arm presentation, several hours were allowed to elapse from the patient's admission before the real state of the case was ascertained. No. 6 (p. 75,) was sent to the hospital at the request of a midwife, who had been attending her, and who, although she assigned no distinct reason, would probably not have taken this step had she not found something wrong. The patient was admitted in the evening; during the whole night she was free from pain, and therefore the labour remained in *statu quo,* and yet the presentation was not ascertained until the following morning, and then by the head midwife, not by one of the medical men. No. 9 (p. 77,) was admitted at seven p. m. with slight pains, the waters having been already discharged; and yet the presentation is not ascertained till eleven. No. 15 (p. 80,) was admitted in a very dangerous state from exhaustion by previous hemorrhage; the membranes were ruptured when she came in at six a.m.; the shoulder was not ascertained to present until three hours after, viz. until the os uteri was nearly fully dilated. The very difficulty of reaching the presenting part ought, we think, to have warned the attendant that all was not right. These omissions could not have happened if due attention had been paid to the nature of presentations and the manner in which the child, during labour, enters and moves through the pelvis and external passages. Again, at p. 103, No. 33 is a case of placenta prævia, where ten days previously there had been a considerable discharge of blood, which had returned shortly before her admission, and yet "no examination was made for seven hours after."

At page 86, No. 37, a case of elbow presentation is given, where turning became impossible from the rigid contraction of the os uteri on the child's body. We cannot help thinking that full bleeding, with a dose of opium afterwards, would have rendered this operation practicable.

On the subject of placenta attached to the os uteri, a number of valuable observations are interspersed with others which are rather commonplace and unnecessary in a work like this; several important points are left quite unnoticed, and upon others we feel it necessary to differ from the author. In estimating the degree of danger from the blood discharged, Dr. Collins (p. 91,) says "there might be internal uterine hemorrhage to an alarming extent." This, in cases of placenta prævia, we can by no means understand. The uterus is filled with liquor amnii, or at least by the child, for the membranes are seldom ruptured until the hand is passed; the blood comes from the os uteri itself, and therefore flows much more readily into the vagina; nor do we exactly see how it is possible for the blood, under these circumstances, to collect in the uterus. In none of the cases to which we have been called, and they are not a few, have we ever seen the slightest trace of internal hemorrhage, nor are we aware of such a case on record. From another observation, a few lines further on, we must also differ. Hemorrhage from placenta prævia does *not* generally come on *slowly;* it usually appears suddenly, although the quantity lost will chiefly depend on the period of pregnancy. In the four last cases which we have attended under these circumstances, the hemorrhage has come on suddenly and profusely. In one case the patient was awoke in the night by a profuse discharge, which soaked through every thing; in two others it burst out with a sudden gush as the patient was standing, without the slightest previous warning; in these two the placenta was centrally attached to the os uteri, and the patient was gone her full time, the hemorrhage was also very profuse.

"Blood lost near the full period of pregnancy," says Dr. Collins, in a note to p. 92, "produces much more debility than in the earlier months. It is very rare to meet with hemorrhage proving fatal before the sixth month of gestation, when properly treated, though often profuse." To the correctness of this observation we fully agree; but why is there no explanation of the fact given, or of these very early cases of hemorrhage from placenta prævia not being dangerous, even although the uterus will not admit the hand. The invaluable observations on this subject in Rigby's Essay on Uterine Hemorrhage ought to have been mentioned, if not quoted. The author remarks, in the same page, "It is also of advantage to observe if the discharge of blood be increased with each labour pain, which is almost invariably the case where the placenta presents; whereas, if it be not so situated, the hemorrhage will at such times be diminished." This is an excellent observation; it is of itself a means of diagnosis, and distinguishes this form of hemorrhage from every other.

We can by no means agree with the author that there are cases of hemorrhage from placental presentation, where the medical man "is obliged either to suffer his patient to sink from loss of blood, or proceed to deliver when the parts are in an undilated and rigid state, in order to afford her the only chance of life." We cannot but express our surprise that the plug should have thus been passed over in silence; a remedy which, in these extreme cases, must be the only means on which our hope of preserving the patient can rest. Besides the observations of Leroux on this subject, does the author forget those of Dr. Burns, Dr. Dewees, or, still more recently, those of Mr. Ingleby in his work on Uterine Hemorrhage? The inestimable value of the plug has been strikingly shown by the latter author, who quotes two remarkable cases where life was preserved solely by this means; one described by Mr. Cusack, in the Dublin Hospital Reports, vol. v. p. 515; the other from the practice of Mr. Grainger, of Birmingham.

The injurious results of not applying the plug are but too strikingly exemplified in No. 34, p. 97, and No. 89, p. 99. The os uteri not being sufficiently dilated, both patients were allowed to sink into a most alarming and desperate state of exhaustion from continued flooding. In one the neck of the uterus was lacerated from endeavouring to pass the hand through its mouth; whereas, if the plug had been used at an earlier period, the patient would have been placed in comparative safety until proper uterine action had come on, and by that time have regained sufficient strength by proper treatment to have enabled her to pass through her delivery with safety. No. 50, p. 101, shows a similar defect.

At page 94, Dr. Collins has noticed a valuable and remarkable fact, which appears to have been scarcely, if at all, noticed by English obstetric writers. "Even a slight injury," says he, "to the mouth of the womb will prove more fatal than an increased loss of blood so long as the strength can possibly bear it." Some explanation of this important fact ought surely to have been given. In what, then, does the danger of slight injury to the os uteri under these circumstances consist; because, in the most favorable labours, nothing is more common than slight laceration of its edge? In placenta prævia, however, it is very different: the os uteri now plays the part of the fundus, and its vessels in such cases undergo nearly, if not quite, the same degree of dilatation and development as those of the fundus are observed to do when the placenta is situated there. A slight laceration of the os uteri now is of very serious consequence: large vessels are torn, which the firmest contractions of the uterus cannot close: hence a constant, though slight, discharge of blood goes on, which gradually exhausts the patient.

"As to whether the hand should be passed through the placenta or at its edge, we must," says Dr. Collins, "be guided by circumstances." We look upon it now as a settled point never to attempt piercing the placenta with our hand. Dr. Rigby has, it is true, advocated this practice, but in so qualified a manner that we should almost doubt if he ever used it at all; for he expressly states that he found it impracticable, "and it ever must be when the middle of the placenta presents to the hand, from the thickness of it near the funis: it must be carefully separated from the uterus on one side, and the hand passed till it gets to the membranes.*" Dr. Collins has overlooked the admirable observations of Dr. Dewees (§ 1,153,) on this subject: they are, indeed, well worthy of perusal, but our limits forbid us to quote them.

"It occasionally happens," (same page,) "that a large quantity of blood collects in the uterus previous to delivery, which, if not removed, keeps this organ distended, and thus creates a tendency to hemorrhage." The meaning of this sentence is obscure. Does the author mean to say that internal uterine hemorrhage can take place in cases of placenta prævia before delivery? The sentence surely belongs to hemorrhage after labour, a totally different subject; and the words "previous to delivery" must have been misprinted for "after delivery:" in no other way is it intelligible to us.

In a case of partial placenta presentation with much hemor-

* Essay on Uterine Hemorrhage, p. 64, 5th Edit.

rhage before and after delivery, (p. 98, No. 77,) would it not have been better practice to rupture the membranes? Uterine action would have increased; the head pressing on the presenting portion of placenta would have stopped the hemorrhage; turning would have been rendered unnecessary; and, labour having followed a more gradual and natural course, the chances of any hemorrhage after delivery would have been much diminished. We have used this plan in at least four cases of partial presentation of the placenta (the os uteri being soft and dilatable,) with complete success. Where uterine action still appeared sluggish, a dose of ergot sufficed to rouse it.

The author's "Observations on Accidental Hemorrhage" are truly practical: they are plain and simple, and worthy of careful perusal. Cordially do we agree with him that Dr. Burns's directions as to dilating the os uteri are "calculated to lead the young practitioner into error;" nor even are they "only justifiable in the most extreme cases of danger," and certainly "never," as he well observes, "until rupturing the membranes has been found to fail in stopping the hemorrhage;" for, if the plug be properly used, the hemorrhage will be controlled, and the os uteri will have sufficient time to dilate, either to let the head pass or to admit the hand. We again regret that Dr. Collins has so completely discarded this valuable aid.

The author has repeatedly observed that the ergot of rye diminishes the action of the heart; thus, for example, at page 134, he says "the mother's pulse, when she got the first dose of the ergot, was 108; at the time the second was given, 102; and, in a quarter of an hour after, it had fallen to 98. This sedative effect I have almost invariably observed the ergot produce, even where it did not seem to act in any other way upon the patient." This is an interesting fact, and deserves attention. We must confess that we have not noticed it.

In his observations on hemorrhage between the birth of the child and expulsion of the placenta, there are no remarks on the effects of portions of placenta being left in the uterus on account of their firm attachment, although, at p. 130, No. 100, a case of this sort occurs. Again, at p. 180, he says, "where the afterbirth is retained by morbid adhesion, it is recommended by most writers to remove as much as can be effected by gentle means, leaving the remainder to be thrown off in the discharges." But is it thrown off in the discharges?—has the author ever observed this fact?—we have not; although on several occasions we have known of cases where considerable portions of placenta were left adherent, and never appeared in the discharges. In the following page he informs us that "in eight cases, hemorrhage occurred in combination with hour glass or irregular contraction of the uterus." No remarks are given on the nature of this irregular distribution of uterine activity. Our own view, viz. that the stricture is produced by the os uteri internum, and not by the body of the uterus, we stated in a former number: it is that which is generally adopted by the first continental authorities.

On the treatment of Hemorrhage after Expulsion of the Placenta, we regret to say that the rules are both scanty and incomplete. Gentle friction on the abdomen, which, in all moderate cases of hemorrhage, we find sufficient to excite uterine contraction, is not even mentioned; but, wherever the uterus is "much relaxed and distended," and the binder and pressure prove insufficient, (which will be almost always the case where there is much uterine inactivity,) the hand is to be introduced. With such practice we cannot coincide. The introduction of the hand is only warranted where the uterus is considerably distended, and, in its present atonic state, unable to expel the coagula: in most of these cases, introducing one or two fingers to the os uteri, and pressing with the other hand on the fundus, is quite sufficient to remove any coagula on the uterus; and we are alluding to cases in our own practice of the most alarming and dangerous description. Instead of applying the binder whilst the uterus shows a tendency to relax, which prevents our feeling the state of the fundus, the great index of our patient's safety, and which, by increasing the warmth of the abdomen, tends still further to relax the uterus, other remedies are to be used. Cold applications are among the most valuable, and yet how insufficient and meager are the observations on this head. "The free admission of air, with the extensive use of cloths soaked in iced or the coldest pump water, will be found useful," is the sum of the author's observations on the external application of cold. It is true he mentions that, in some very obstinate cases, he has poured cold water from a height upon the abdomen with success, as if the fact were new. He draws no practical inferences from it; and, from talking of introducing snow and ice into the vagina, is evidently ignorant of the inestimable principle laid down by Dr. Gooch, that "*cold applied with a shock is a more powerful means of producing contraction of the uterus than a greater degree of cold without the shock.*"

With the use of injections of cold water, &c. in the vagina, he professes to have no experience, but "should rather think it not advantageous." Yet we consider this as actually one of the most powerful and valuable means we possess of inducing uterine contraction in dangerous cases. An equally valuable remedy here, viz. the ergot, is not mentioned; nor the application of the child to the breast.

We must confess ourselves greatly disappointed with the author on this subject. His after-treatment is judicious and simple, except on one point. "Severe and acute pain of the temples very frequently succeeds profuse hemorrhage, in a shorter or longer time after delivery: in relieving which, we have almost constantly found the application of eight or ten leeches to the affected part successful." This pain of head, which is occasionally one of the most oppressive and severe forms of headach known, results from debility and want of due circulation in the brain: how can this be permanently or safely relieved by leeches? A dose of camphor and extract of henbane, repeated according to circumstances, has rarely if ever failed to remove it; and it is a far safer form of sedative than mere opium. The subject is of such intense interest that we could have wished to enter much more fully upon it, but our limits forbid.

In a case of hemorrhage after birth of the child, (p. 158, No. 125,) the patient was fifty-nine hours in labour, the head pressing firmly against the ischia for some time, with evidences of serious pelvic deformity; yet no assistance was rendered until the fœtal heart ceased to be heard; no bleeding was practised, although the pulse was between 120 to 130, and the external parts were œdematous. No attempt was made to apply the forceps; nor was the head perforated, until it had remained stationary, pressing against the ischia for nearly twenty-four hours. The consequences, we need hardly add, were dreadful sloughing and gangrene of the vagina, communication between it and the rectum, and death on the ninth day.

The author's observations on not hurrying labour, as being a powerful means of preventing retention of the placenta, are good. In speaking of the cases where the placenta was retained, he says "in four the delivery had been forced." We strongly object to this expression: we do not *force* delivery in the present day.

We agree with Dr. Collins in saying "Puerperal convulsions occur almost invariably in strong plethoric young women with their first children, more especially in such as are of a coarse make, with short thick necks;" but we complain that no discrimination is made between the different forms under which these convulsions appear. Dr. Dewees's excellent observations on this subject have not excited the author's attention, or he would not have denied "that puerperal convulsions were caused by the irritation produced in the dilatation of the mouth of the womb;" he would have been aware that convulsions arising from this cause are preceded and accompanied by symptoms of strong determination to the head; that there are other convulsions, arising from a totally opposite state of the circulation; that there are others where the cerebral circulation is in an apoplectic state; and lastly, others which partake strongly of the hysteric character. He would not have waited three hours after perforating before attempting to extract the child, knowing that *its* presence in the uterus and passages was the exciting cause of the convulsions: he would have effected the extraction with ease, as the pelvis was well formed. The forceps slipped with the second child, which had thus to be perforated also. The mother was given a bolus of calomel and jalap and one grain of opium, the bowels being "most obstinately torpid." She died in thirty-four hours.

The next case of convulsions shows equally objectionable practice, (p. 203, No. 8.) The first fit came on whilst the head "was pressing on the perineum and the pains strong." It was allowed to remain in this state for *eight* hours, without any assistance being given, when, all the symptoms of rupture of the uterus coming on, the child was delivered by perforation. The patient died, and the dissection showed extensive rupture of the uterus and vagina.

The next case is even worse, and shows the impropriety of allowing the patient to be in labour without making a proper and early examination. The patient was allowed to be *twenty-four hours* in labour, during which convulsions, and ultimately fatal rupture of the vagina, took place, before the real state of the head (hydrocephalus of great size,) was ascertained.

At pages 207 and 208 are the records of two fatal cases of convulsions, viz. Nos. 21 and 25, where, in our opinion, assistance was also delayed too long.

The use of tartar emetic, which the author recommends, p. 212, is very serviceable in *some* cases of convulsions, especially when connected with a neglected state of the bowels, but not in every case. His note to the next page, respecting the use of camphor, and also to page 227, on the exhibition of opium, seem to show a want of discrimination of the cases where these remedies were indicated and where they were not.

At page 223, No. 20, is a case of convulsions, where the attack came on when the os uteri was "fully dilated, the pains vigorous, and the head advancing:" no assistance was given, and the patient was not delivered till five hours after of a dead child, the heart of which had been distinctly heard an hour before. The author states that "the case was not such as to admit of the application of the forceps." The reasons for the non-indication of the forceps should have been given: she had been freely bled, the bowels "well acted on," and passages well dilated; what was there to prevent the forceps being applied? No. 11, in the next page, was allowed to be eight hours in labour after the appearance of the convulsions without assistance, although "the os uteri and soft parts were well dilated, the pains strong, and the head making considerable progress."

In speaking of the cautions in order to avoid a recurrence of the attack in a subsequent pregnancy, Dr. Collins very justly remarks that "a severe paroxysm of genuine puerperal convulsions is seldom met with in any individual who has *before* laboured under such a seizure." This is an excellent remark, and he applies it in the next page (238,) with equal justice to puerperal mania. Dr. Gooch's excellent observations upon this subject confirm the fact.

On Rupture of the Uterus, Dr. Collins correctly observes, that "the part which usually gives way is at the junction of the cervix uteri with the vagina :" he also adds, "either anteriorly or posteriorly, and, according to my experience, equally in both situations." The opinion hitherto was, that it occurred most frequently at the *back* part; but we freely confess ourselves inexperienced upon this subject.

We are surprised to find thirty-four cases of rupture; (about one in every 480 labours,) a proportion truly awful, and we confidently assert neither equalled nor approached in the practice of any similar institution. We should have been at a loss to account for this extraordinary number, had not the melancholy details of these deplorable cases given us but too sufficient an explanation; and, whilst we admire the author's candour in thus freely stating them, we must nevertheless reprobate in the strongest and most unequivocal manner the practice under which many occurred. We would gladly have avoided entering into the details of these sad cases; but we cannot pass on without showing the grounds upon which we have made these observations. We can only do this by calling the reader's attention to the chief points of those cases, the management of which we object to.

P. 259, No. 19, her first labour, "the head remained for some time high up;" it gradually descended, and in nineteen hours, when symptoms of rupture appeared, it was low in the pelvis; "the pressure on the urethra was so considerable, that there was some difficulty experienced in passing the smallest sized flexible catheter. The head was lessened, but not delivered without the greatest exertion;" and even afterwards some force was necessary for the extraction of the shoulders. Ought not the fact of the head remaining so long high up in the pelvis of a primipara to have been sufficient warning that there was want of room? If an early examination had been made with due care and attention, it is almost impossible that the unusual manner in which the head was pressing upon the brim could have escaped attention. After all, the patient scarcely seems to have died from the effects of the laceration, which was all but healed, but rather exhausted by the extensive sloughing and ulceration in the soft parts produced by this delay in giving assistance.

Should a patient who (P. 291, No. 16,) had been "force delivered," as Dr. C. calls it, before in the hospital, to go four hours after the full dilatation of the soft parts, with the head low down, the outlet of the pelvis evidently narrow, without giving assistance? Rupture ensued.

The next case (No. 18,) shows equally objectionable practice: the woman was in labour from the preceding day; "the head was high in the pelvis, and made but little progress; the soft parts were well dilated, pulse between 120 and 130; tongue foul. She continued in this state for some hours:" symptoms of rupture set in; the head, being out of reach of the forceps, was lessened. Can we justify allowing a patient to remain in labour with the head in this situation, with such a pulse and tongue, "for some hours?" Mischief *must* inevitably ensue. The dissection showed that the injury had resulted from the vagina being squeezed between the head and a projecting portion of sacrum. We can scarcely agree with the author in thinking that the injury had taken place before admission.

P. 299, No. 28. "A feeble delicate woman was thirty-six hours in labour previous to the setting-in of the above symptoms (of rupture,) and had been force delivered in this hospital eleven months ago." With such a confession, what can justify a delay of thirty-six hours?

P. 300, No. 32, was admitted in the evening with most dangerous symptoms of exhaustion, having been sent "many miles from the country in severe labour;" and yet (would the reader credit it?) she was not delivered until the next morning at nine o'clock, *as the child's heart had ceased to beat,* and the patient was in *articulo mortis.* Is *this* what we are to understand by the tenderness of not choosing to perforate the head of a living child,—waiting until it had been gradually destroyed by the length and severity of the labour, and the mother brought into a dying state? If, by means of the stethoscope, the having ascertained at one period of her labour that the child was alive, led to such deplorable practice, we can but regret the author having ever used it.

P. 301, No. 3, shows in the strongest manner the great impropriety of not having a properly qualified person to examine every patient on admission. The patient was admitted at nine *p. m.,* in labour of her first child. An elbow presented, which was actually mistaken for a knee, and she remained without any further notice for six hours. The real nature of the presentation was now ascertained: the body had become closely wedged in the pelvis; there was *extreme* pelvic deformity, (an antero-posterior diameter of only two and a half inches.) Turning was now impossible. The thorax was perforated. "It required most laborious exertion for two hours and a half to complete the delivery, which was only accomplished by taking the child in pieces." She died in four hours. Extensive laceration of the cervix uteri and vagina was found on the right side. How much suffering and difficulty might have been avoided if the real nature of the case had been properly ascertained before the presenting part had become so firmly impacted in the pelvis. If any parts of the child liable to present can be distinguished from each other, certainly it is the knee from the elbow: the difference cannot be mistaken. But, even putting aside this oversight, how was it possible to avoid noticing such unusual pelvic deformity? If this "person in attendance" was a pupil, he must indeed have been a beginner, and, as such, ought not to have been intrusted so entirely with a case. If it was a midwife, most assuredly she ought to have been dismissed as unfit for such an office. Further comments on these cases we must decline making.

"The operation of turning," says our author, p. 242, "is not unfrequently a cause of laceration of the vagina or mouth of the uterus," &c. That it may prove a cause, he has shown in at least three cases; but that laceration is "not unfrequently" thus produced, we cannot admit,—at least on this side the water.

It is with pleasure we turn from this subject, (upon which we have unavoidably dwelt much longer than we could have wished,) to Dr. Collins's excellent "practical observations on Twin Births:" they are plain and judicious. The placentæ of twins are (as he rightly observes,) generally united, and not separated, as has usually been asserted; the two circulations, we believe, however, are always distinct. The following observation is very valuable:

"When we reflect," says Dr. C., p. 313, "as has been computed, that the ordinary proportion of deaths in women giving birth to twins is one in twenty, whereas, with single children, a death does not occur in nearly five times that number, we cannot but see the necessity for the most scrupulous attention on our part. It is no matter what the nature of the attack may be, whether hemorrhage, convulsions, fever, &c., it will be found much more dangerous in women giving birth to a plurality of children than others."

After inculcating such really valuable rules, we cannot but be surprised to see his practice at p. 318, No. 174. After the patient (first pregnancy,) had been forty hours with the first, why could not the second child be delivered with the forceps? Why allow the head, which was low in the pelvis, to remain there eight hours? We cannot understand why "the case did not admit the application of the forceps:" the head had "descended into the pelvis," and, of course, the parts were well dilated by the passage of the first child.

In his observations upon Prolapsus of the Cord, the author has given us no remark whatever on the circumstances under which this displacement occurs; which is to be regretted, because, in a work like the present, (a record of great experience,) such observations are always valuable.

He almost entirely discards turning the child, or the application of the forceps. We must confess we have not found turning so dangerous to the mother as he considers it to be, and should scarcely think ourselves justified in not undertaking this operation where the cord was felt pulsating strongly, and the os uteri and passages were in a favorable state. Trying to keep up the cord with the fingers, or with a piece of sponge, ought not even to have been attempted at all; its utter uselessness has long since been proved. The author proposes to attach the cord to the end of a flexible male catheter, and thus pass it up above the head, leaving the catheter there during the labour; but has given no results of his trials. An interesting paper on this very subject was published last year by Dr. Michaelis, of Keil, in the third volume of the "Neue Zeitschrift für Geburtskunde," where he succeeded in replacing the cord above the presenting part in a considerable number of cases with perfect success. His plan is as follows: he passes a piece of twine doubled through a flexible male catheter, so that the two ends hang out at the lower extremity, and the loop at the upper or vesical extremity; having introduced the stilet, and passed the loop round the cord, he withdraws the stilet just so much that, by bending back the point of the catheter, the end of it may project through the orifice. Upon this he hooks the loop, and pushing up the stilet to its full extent, and drawing down the ends of the ligature at the lower extremity, the funis is thus firmly attached to the upper end of the catheter. Having now carried it as high as possible above the brim, he withdraws the stilet; the funis is instantly disengaged from the catheter, which is therefore removed, leaving the funis behind.

The author (p. 350,) quotes the following passage from Dr. Denman: "When the child is dead, all the efforts of art must be useless to it, and may be injurious to the mother: we must therefore be satisfied with permitting the labour to proceed as if the funis had not descended;" an opinion, as he confesses, supported by Burns and other authorities, but from which he differs, not seeing any advantage in allowing the patient to remain hours in labour, when delivery may be quickly accomplished by lessening the head. The author mistakes Dr. Denman's meaning. By "permitting the labour to proceed as if the funis had not descended," Dr. D. merely meant that he should treat it as a common natural labour; but assuredly he would never have allowed it to go on thirty-six, forty-eight, or even more hours, as reported in several of the author's cases. The quotation, both at its beginning and end, shows (to our mind) better practice than what the author has advocated: it shows that, where the child was alive, Dr. Denman would probably have interfered with art; and that, where it was dead, and the interference of art not indicated on the part of the mother, he would have left it to nature. This, in fact, was his practice; and it was judicious. The author's tabular view of these cases is incomplete, from not having the presentation of the child noted, which would have greatly added to its interest and value.

On the subject of Sloughing of the Urethra, &c., Dr. Collins considers that the symptoms threatening this mischief do not indicate lessening the head so long as the child is alive. We should feel inclined to modify this rule considerably. Where the head has been some time pressing against the symphysis pubis without entering the pelvic cavity, and there is no chance of being able to deliver with the forceps, we should consider the perforator indicated, without waiting until the child had died from the effects of the labour; for by this time we should run a considerable risk of losing the mother also. We hold that, where there are not evident indications for the perforator, it can rarely happen that the head is so long pressing on the soft parts as to produce sloughing, and yet the child be alive, without the forceps having been indicated.

We fully agree with the author's observations respecting the Varieties of Puerperal Fever. "The extreme difference of opinion,"

says he, p. 390, "and the very opposite measures recommended by practitioners, arises chiefly, I am satisfied, from their treating of every variety of puerperal fever as one and the same disease; whereas, there is perhaps not any other which exhibits a greater diversity of character in different situations, and even in the same situation at different periods." If this fact had been properly attended to, we should not have seen so discrepant and opposite opinions in the various works on this subject. The form of the disease which he has described appears in most cases to be very similar, if not identical, with the common form observed in London, beginning with pain in the region of the uterus, which is felt large and hard, and tender upon pressure, sometimes extending to one side; the pain, in the course of a few hours, extends over the whole abdomen, which becomes tympanitic; the pulse is rarely hard, although quick and perhaps sharp; the tongue is mostly covered with a thin white fur, which, if the symptoms grow more unfavorable, becomes dry and brown.

"The result of my observations upon the treatment of puerperal fever," says Dr. Collins, p. 396, "is, that *general* bleeding, except where there is a strong, full pulse, and the symptoms are of a high, inflammatory character, is injurious. On the contrary, *local* depletion, by the application of three or four dozen leeches, followed by the warm bath and stuping, all of which should be repeated according to circumstances, as often as the strength will permit, seemed most beneficial. These means, together with the active employment of calomel conjoined with hippo and opium, aided by mercurial frictions, offer the best prospect of relief. Blistering the entire abdomen, after leeching had been pushed as far as could be, was found serviceable. In some instances the debility from the very commencement was so excessive as to induce us to apply the blister at once, using calomel and stimulants at the same time."

Dr. Collins's views on the use of the lancet in these cases are good and valuable: they are cases which are very far from admitting the indiscriminate use of bloodletting. The combination of calomel with ipecacuanha ("*hippo*,") is very excellent, and we consider that in many of the recorded cases it proved of eminent service; but we feel equally certain (and not from slight experience,) that, if it had been combined with opium in the form of Dover's powder, it would have answered much better. We have, moreover, strong reason to believe that, if the first dose of calomel had been one of eight or ten grains, combined with an equal quantity of Dover's powder, or four grains of pulv. antimonialis, according to the character of the pain, state of the bowels, &c., and not repeated for some hours, that the effect produced would have been more certainly beneficial, and that a much less quantity of calomel would have sufficed. We cordially agree with the remark of the author, in a note to p. 396, "it is supposed by some practitioners that, when we can get the system under the influence of mercury, recovery is *certain*. This is not the fact, as I have seen several cases where death took place under these circumstance: it is, notwithstanding, a very favorable occurrence." Where he has given large doses of calomel, we regret that so little moderation was used: scruple doses of calomel every two hours is a plan of treatment almost sufficient to anticipate the disease. To his whole series of external applications we feel compelled to object. The hot "*stupes*" (Anglice, fomentations,) relieve the patient for a time; but how can we be sure of their being regularly renewed every two or three minutes, for an hour or two, by a nurse? They generally do more harm than good: the patient is made thoroughly wet and uncomfortable, and the flannels are frequently allowed to remain on long after they have lost their heat. We feel convinced that, in several of the cases recorded by the author, the obstinate return of the abdominal pain was as much the result of this as of any other cause. The only safe and effectual manner of applying heat is by folding a large poultice of linseed meal spread flat in a linen cloth, and covering the whole abdomen with this as hot as the patient can bear it with comfort. When well made, it will retain its heat for four, five, or even more hours, and the beneficial effects produced fully warrant the high encomiums which Dr. Gooch has passed upon it. The other external applications, viz. blisters, mercurial inunction, &c., we have seen applied, and are strongly inclined to hold them in no cases superior to the poultice when properly made, and in many cases far inferior.

To our great astonishment, the whole subject of Puerperal Fever is passed over without the slightest attention being paid to the state of the lochia and vaginal discharge: the word *lochia*, as far as we can perceive, is not even mentioned. We are at a loss to explain the cause of such a singular omission of all notice of the state of a discharge, the regular and healthy secretion of which is of such vital importance in these cases. Of course, therefore, the frequent syringing the vagina with warm water is equally unnoticed, a plan which we have used extensively with the most successful results, and which, independently of its value in washing away any putrid coagula or lochia which may have collected in the vagina, (a source of irritation of the very worst kind,) is of the greatest utility as an internal fomentation to the uterus; frequently bringing on a return of the lochia which had been suddenly checked at the commencement of the attack, and producing a degree of relief not less complete than striking.

The trial which Dr. Collins has given of the oil of turpentine in his practice is certainly not more in its favour as a remedy in this form of puerperal fever: in several cases it appears distinctly to have aggravated the abdominal pain. Whether it was to this cause, or the "stuping," or inattention to the vaginal discharge, or more or less of each together, we know not; but we have scarcely ever seen so little relief produced by general and local bleeding, and the use of calomel and ipecacuanha or opium, as here, although they were judiciously and decidedly employed by the author. We cannot help thinking that these remedies would have acted more successfully if aided by a large hot poultice as above described, by warm vaginal injections, or by a milder and less stimulating purge than turpentine. It would be, nevertheless, unjust towards the author to suppose that the majority of the fatal cases of puerperal fever would have terminated otherwise, even if those alterations in practice which we have suggested had been adopted. Many of them were cases which, from the beginning of the disease, gave little chance of success: still, however, many were cases of a milder form, which might have justified the hope of a more favorable issue. We are surprised that the author has not found the inexpediency of giving jalap, senna, and such like griping purges shortly after labour. We have seen such repeated instances of an attack of abdominal pain succeeding the exhibition of these remedies, and so many relapses from this cause, that we carefully avoid giving this class of laxatives; confining ourselves to castor oil, carb. and sulp. of magnesia, in aq. menth. pip. &c.

Dr. Collins's arrangements for the prevention of this dreadful malady are truly admirable, and do the highest credit to his activity and judgment. Although we have nearly reached our limits, still we cannot alter the original description. Puerperal fever prevailed at the Dublin Lying-in Hospital in February, 1829.

"We had," says Dr. Collins, "all the wards in rotation filled with chlorine gas in a very condensed form for the space of forty-eight hours, during which time the windows, doors, and fire-places were closed, so as to prevent its escape as much as possible. The floors and all the woodwork were then covered with the chloride of lime, mixed with water to the consistence of cream, which was left on for forty-eight hours more. The wood-work was then painted, and the walls and ceilings washed with fresh lime. The blankets, &c. were in most instances scoured, and all stoved in a temperature of between 120 and 130°.

"From the time this was completed until the termination of my mastership in November 1833, we did not lose one patient by this disease. As the wards of the hospital are occupied by the patients in rotation, as soon as each in succession was vacated, I continued the use of the chloride of lime, confining its application to the floors. In this way each ward was washed every ten or twelve days, the solution being left on for twenty-four hours, during which time the blankets, quilts, linen, &c. were suspended, so as to be exposed completely to the chlorine gas, which is copiously disengaged from the preparation mentioned. The chloride of lime was then carefully washed off, and the boards, when dry, polished with a brush. It may appear strange that a process such as stated should be considered advisable in an establishment which is at *all times* kept in the most perfect state of *neatness* and *cleanliness* in every respect; so much so, that few private houses would bear comparison; yet the result consequent on such a practice will fully justify our having had recourse to it. To the ventilations of the hospital, I have always paid the most strict attention, so that no heated or vitiated air might be suffered to accumulate.

"In addition to the air tubes, &c., as described when treating of trismus nascentium, I had from one inch and a half to two inches of the upper sash of the window most remote from the patient's bed kept open day and night, throughout the entire year, except during extreme cold at night in winter; thereby ensuring a free circulation of pure air.

"All the beds in the hospital are composed of straw; nor is any one used more than a second time without the cover having been washed and the straw renewed. In every instance where the patient *dies*, this is at once done, and, should the most remote symptom of fever have been present, every article connected with the bedding is instantly scoured and stoved; the wood-work and floor washed with the chloride of lime solution, and the entire ward whitewashed : this is readily effected, as the sick are invariably placed in a small ward apart from the healthy. To this precaution too much attention cannot be paid. I am satisfied the *instant* separation is of vast importance to both."

We have but insufficiently treated this important subject; and there are several other points of interest to which we could have devoted some observations; we must, however, extract a case from the next chapter, as coming under the present subject of puerperal fever, and as giving an instance of a peculiar and very dangerous form of this disease.

No. 639, p. 473. "She was delivered on the 13th of February: on the 15th she complained of tenderness of the abdomen, which was removed by leeches and stuping. On the 16th, she suffered from uneasiness in her stomach; and, on the morning of the 17th, her pulse sunk rapidly, and her extremities exhibited in the most marked manner the appearances of diffuse cellular inflammation, particularly the right fore-arm; her strength continued to fail, and she died the same evening, although stimulants and cordials were diligently employed. On dissection, the abdominal viscera appeared healthy ; there was a slight blush of redness on the anterior surface of the uterus; the muscles of the body were in a remarkable state of decomposition, particularly those of the right fore-arm, where they appeared in a state of putrefaction. The blood was fluid in all parts of the body."

In our own experience this has been the most fatal species of puerperal fever which we have met with: it differs essentially from the other and more common form. The uterus is seldom affected; there is little or none of that hardness, swelling, and pain of this organ, which is seen in the other form. The pulse is rarely under 130; mostly 140, small, and weak, (at times however hard); the tongue red, streaky, and dry; in some instances great confusion of head; constant vomiting of greenish mucus; tympanitis coming on rapidly, without the usual degree of abdominal pain; the sudden collapse, with cold tongue and extremities; dusky appearance of the face, and large patches of livid ecchymosis upon the extremities. One of the most singular features of the disease is the enormous collections of ill-conditioned pus, forming in different parts of the body, especially in the muscles of the extremities. An indurated painful spot is felt, for example, in the muscles of the fore arm; a spot more frequently observed by us to be affected than any other part. The extremity becomes stiff and immoveable; the induration increases in extent; fluctuation follows; if the patient lives long enough, an immense collection of pus is formed. Perhaps it can scarcely be called pure inflammation of the cellular tissue. On examining after death extremities of this sort before fluctuation had commenced, the whole cellular tissue appeared in a half-dissolved state; the muscles firm to the feel, but easily torn through; their natural colour entirely changed for a dirty ashy hue, more like boiled meat than anything else. The fluid state of the blood as noticed by Dr. Collins is also a remarkable fact; the blood in those cases which have come under our notice resembled considerably thin claret in appearance. The disease appeared to consist very much in a morbid state of the circulating fluid, and the relief afforded by alkaline and saline remedies seems to confirm this view.

At P. 476, No. 745, we are sorry to meet with another instance of what appears to us highly objectionable practice. "The patient was admitted in a high state of fever," p. 120, "tongue loaded, teeth covered with sordes;" it was her first labour; "the urine was three times removed by the catheter, and, on its last introduction, a considerable quantity of bloody fluid came away," and yet she was allowed to remain in this state twenty-six hours before art was employed to deliver her. No bleeding or other treatment is mentioned. We cannot imagine any reason for keeping this poor creature in severe suffering until her almost moribund state gave warning that she would die undelivered, if left any longer to herself: as it was, "she was delivered on the 12th of August, and lingered until September 3d, when she died."

It seems to us a matter of surprise that, in cases requiring the crotchet, and where the mother was delivered artificially in her former labours, artificial premature labour was not thought of. Nos. 1005 and 1032, p. 480, are instances of this: the first patient had only one child alive, (her seventh,) having been artificially delivered in her previous labours; in the other case, it was the "*fourth* time that she had been delivered *artificially*. Fifteen months since she was delivered in this hospital with the crotchet." Artificial premature labour is not even hinted at by the author, yet, in our own experience, it has been attended with the happiest results.

The next case (No. 1038,) we must be allowed to give verbatim; the reader will then be better able to judge for himself concerning it.

"This patient was admitted in labour of her first child. Uterine action was feeble, and continued so for seventy-two hours after she came in. As the fœtal heart had ceased to act for some time, and the pulse became hurried, it was considered advisable to deliver her. The os uteri was not fully dilated; the head was high, and resting above the pubes; it was lessened, and cautiously brought down with the crotchet. Severe abdominal inflammation set in shortly after delivery, which resisted the most prompt and active treatment, and proved fatal on the sixth day." (P. 481.)

*As* the fœtal heart had ceased to beat for some time, and her pulse was becoming flurried, Dr. Collins thought it advisable to deliver her. This has been his rule nearly, if not entirely, throughout the work: its results we have already shown. But, supposing the fœtal heart had *not* ceased to beat, in order to let him perforate, what was to be done then? Wait seventy-two hours more;—i. e. if the mother had lived so long? "The os uteri was not fully dilated, the head was high and resting on the pubes." This being the case in a primipara, even for twenty-four hours, ought to have given ample proof of a narrow pelvis. This, and the many other similar cases which we have commented on, do not exactly confirm Dr. Collins's assertion at p. 2, that no useful information is to be gained by examination in the early stages of labour. The pelvis was an unusual one; it was evidently the "pelvis æqualiter justo minor" of the German schools, being three and a half inches from pubes to sacrum, and four and a half transversely.

Dr. Collins's practice among the children appears to have been very successful, and we regret that we have been favoured with scarcely any details of infantile management at this early and interesting period. We must refer the reader to a quotation respecting the mortality of children at the end of our statistical observations.

The *Trismus nascentium* appears to have been a most prevalent and fatal disease in the hospital in former years. The author quotes a passage from Dr. Joseph Clarke, wherein he states that "at the conclusion of the year 1782, of 17,650 infants born alive in the hospital, 2,944 died within the first fortnight."

"That is," says our author, "nearly every sixth child, or about seventeen in the hundred: about nineteen of every twenty of these, he adds, died of *nine-day fits*. He considered a foul and vitiated state of the air in the wards of the hospital to be the principal cause of this disease: to counteract which, he had apertures of considerable size made in the ceilings of each ward, (these have been since changed for air-tubes, six inches in diameter, passing to the roof;) three holes of an inch diameter were made, in an oblique direction, through each window-frame at top; the upper part of the doors opening into the galleries were also perforated with numerous holes. By these means a free circulation of air was at all times secured through the wards, and effected in such a way as put it out of the power of the nurses to control it. The consequences of these alterations, as stated by Dr. Clarke, were favorable beyond the expectation of the most sanguine. Of 8,033 children born subsequently to the wards being ventilated as described, only 419 died; that is, about one in nineteen and three-quarters, or from five to six in one hundred, instead of the enormous mortality before mentioned."

With these quotations we close our review of Dr. Collins's work. That it has been carefully analysed, we trust is proved by our observations; that we have acted impartially, we hope is also shown by our readiness, nay even anxiousness, to give the author the full meed of praise, wherever it was due. We have, it is true, differed from him essentially on certain points of his practice; so much so, as to censure it here and there in the most unequivocal manner. We should not have acted fairly to ourselves, or to our obstetric brethren of Great Britain and Ireland, to have tacitly approved of these portions of his practice, by passing them by without full and sufficient comment. Still, as a whole, the work is very creditable to the author. As a statistical record, it will be always useful and interesting; but we cannot forget how much more might have been made out of such an ample field for observation and enquiry.

---

